# Supplementary material for: AhR diminishes the efficacy of chemotherapy via suppressing STING dependent type-I interferon in bladder cancer
Source: Nat Commun. 2023 Sep 5;14:5415. doi: 10.1038/s41467-023-41218-5 (PMC10480448; doi:10.1038/s41467-023-41218-5)
Supplement: Supplementary file 1 — Supplementary Information [file 41467_2023_41218_MOESM1_ESM.pdf]

Supplementary Information

AhR Diminishes the Efficacy of Chemotherapy via Suppressing STING Dependent Type-I Interferon in Bladder Cancer

Ma et al.

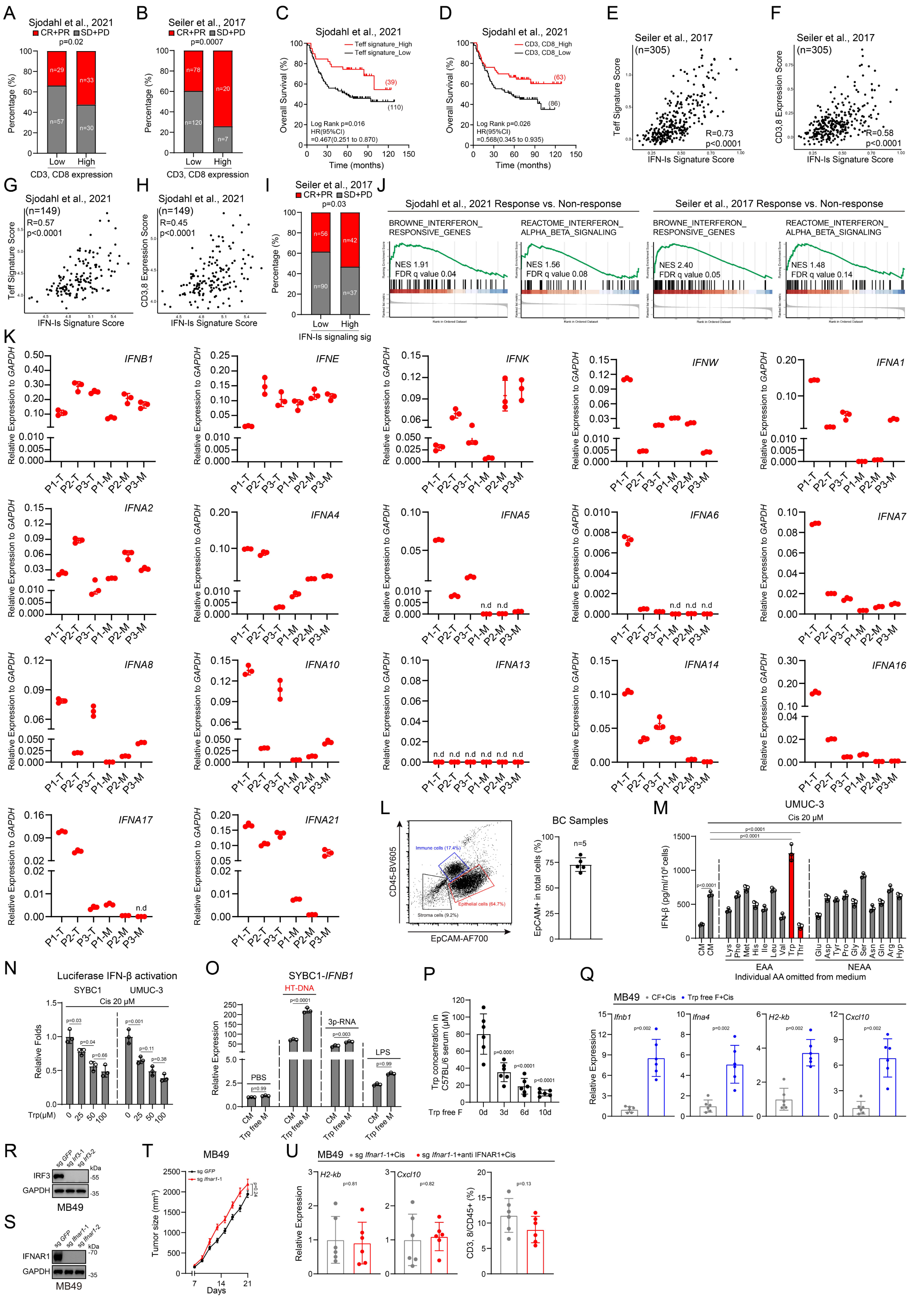

Supplementary Fig.1 Trp diminishes IFN-I production induced by cisplatin, related to Fig.1.

A, B The correlation between NAC response and CD3, CD8 expression. CR=complete response: PR=partial response; SD=stable disease; PD=progressive disease[1, 2]. **C, D** Kaplan-Meier survival curves of patients with low and high Teff signature score or CD3, CD8 expression score[1, 2]. **E-H** The correlation between IFN-Is signature score and Teff signature score or CD3, CD8 expression score[1, 2]. **I** The

correlation between NAC response rate and IFN-Is signature expression[2]. **J** GSEA plot shows upregulation pathway genes in NAC response samples (CR+RP) compared to non-response samples (SD+PD)[1, 2]. **K** mRNA expression of indicated IFN-Is in paired tumor and myeloid cells isolated from treatment naïve bladder cancer samples. **L** Proportion of immune cells, stroma cells, and epithelial cells in bladder cancer samples (n=5). **M** ELISA for IFN-β content in the supernatant after individual AA omitted from medium and treated with cisplatin. **N** Luciferase reporter assays for IFN-β after Trp-free medium culture and treated with cisplatin. **O** mRNA expression of *IFNB1* after treated with HT-DNA, 3p-RNA, or LPS under Trp-free medium culture. **P** HPLC-MS detection of Trp levels in mice serum after Trp free feed (n=6). P-value compared to 0d. **Q** Effect of Trp-free feed combined with cisplatin on mRNA expression of *Ifnb1*, *Ifna4*, *H2-kb*, and *Cxcl10* in tumor (n=6). **R** Immunoassay for *Irf3* knocked-out efficiency. **S** Immunoassay for *Ifnar1* knocked-out efficiency. **T** Effect of *Ifnar1* knocked-out on MB49 growth (n=6) (Mean ± SEM). **U** Effect of *Ifnar1* knocked-out combined with anti-IFNAR1 neutralizing antibody and cisplatin on mRNA expression of *H2-kb*, and *Cxcl10* or percentage of $CD8^+$ T cells in tumor (n=6). P-value by two-tailed Pearson's chi-square test **(A, B, I)**. P-value by Kaplan-Meier survival analysis **(C, D)**. The correlation coefficient and two-tailed p value were calculated using Pearson's correlation analysis **(E-H)**. P-value by One-way ANOVA **(M-P)**. P-value by two-tailed Wilcoxon **(Q, T, U)**. Area under curve (AUC) was used to determine the optimal cut-off. All p-value < 0.05 as statistic difference. Error bars represent Mean ± SD, unless otherwise indicated. Three biologically independent experiments were performed **(K, M-O, R, S)**. Source data are provided as a Source Data file.

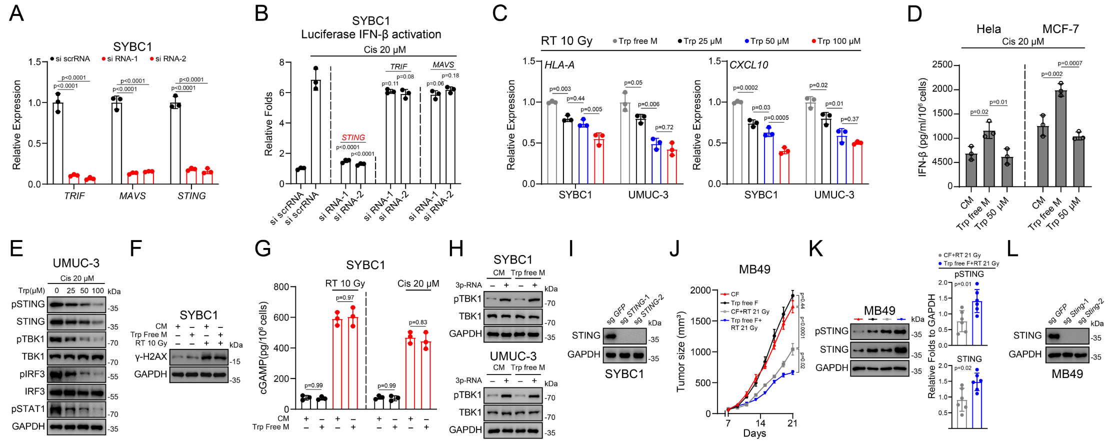

Supplementary Fig.2 Trp suppresses IFN-I production at STING level, related to Fig.2.

A mRNA expression for indicated genes knocked-down efficiency. **B** Luciferase reporter assays for IFN-β after knocked-down indicated genes and treated with cisplatin. P-value compared to si-scrRNA group. **C** mRNA expression of *HLA-A* and *CXCL10* after Trp-free medium culture or Trp supplementation and treated with RT. **D** ELISA for IFN-β content in the supernatant after Trp-free medium culture or Trp supplementation and treated with cisplatin. **E** Immunoassay for representative cGAS-STING related genes expression after Trp supplementation and treated with cisplatin. **F** Immunoassay for H2A.X expression after cultured in CM or Trp-free medium and treated with RT. **G** ELISA for cGAMP content cultured in CM or Trp-free medium and treated with cisplatin or RT. **H** Immunoassay for pTBK1/TBK1 expression after cultured in CM or Trp-free medium and treated with 3p-RNA. **I** Immunoassay for *STING* knocked-out efficiency. **J** Effect of Trp-free feed combined RT on MB49 growth (n=6) (Mean ± SEM). **K** Immunoassay of pSTING and STING expression in MB49 bearing mice after indicated treatment (n=6). **L** Immunoassay for *Sting* knocked-out efficiency. P-value by One-way ANOVA **(A-D, G, J)**. P-value by two-tailed Wilcoxon **(K)**. All p-value< 0.05 as statistic difference. Error bars represent Mean ± SD, unless otherwise indicated. Three biologically independent experiments were performed **(A-I, L).** Source data are provided as a Source Data file.

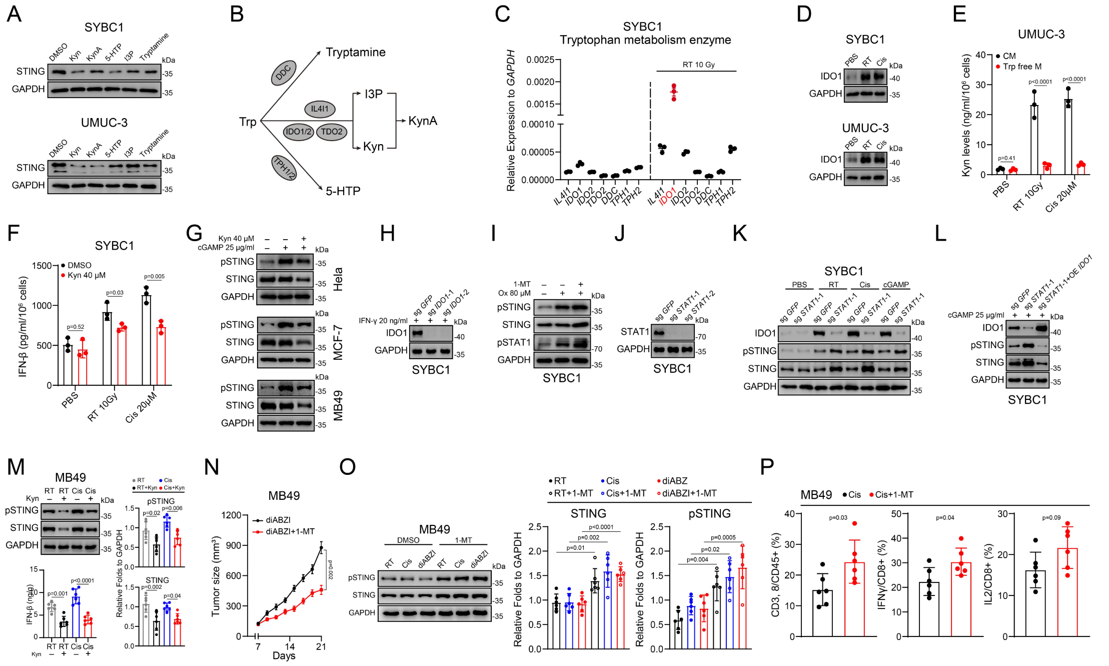

Supplementary Fig.3 Indoleamine 2,3-dioxygenase 1 (IDO1)-mediated Trp decreased STING level and IFN-I production, related to Fig.3.

A Immunoassay for STING expression after treated with indicated endogenous Trp-derived metabolites. **B** Diagram shows Trp metabolites and corresponding metabolic enzymes. **C** mRNA expression of Trp catabolic enzymes after treated with RT. **D** Immunoassay for IDO1 expression after treated with cisplatin or RT. **E** HPLC-MS detection of Kyn levels after treated with cisplatin or RT under indicated medium. **F** ELISA for IFN-β content in the supernatant after treated with cisplatin or RT combined with Kyn. **G** Immunoassay for pSTING/STING expression after treated with cGAMP or cGAMP combined Kyn. **H** Immunoassay for *IDO1* knocked-out efficiency. **I** Immunoassay for representative cGAS-STING related genes expression after treated with oxaliplatin combined with 1-MT. **J** Immunoassay for *STAT1* knocked-out efficiency. **K** Immunoassay for IDO1, pSTING, and STING expression of *STAT1* knocked-out cells after indicated treatment. **L** Immunoassay for IDO1, pSTING, and STING expression of *STAT1* knocked-out cells or *STAT1* knocked-out with overexpress *IDO1* cells after treated with cGAMP. **M** Immunoassay for pSTING and STING expression and IFN-β content in MB49 bearing mice after indicated treatment (n=6). **N** Effect of diABZI combined with 1-MT on MB49 growth (n=6) (Mean ± SEM). **O** Immunoassay of pSTING and STING expression in MB49 bearing mice after 1-MT combined indicated treatment (n=6). **P** Percentage of $CD8^{+}$ T cells, $IFN\gamma^{+}CD8^{+}$ T cells, and $IL2^{+}CD8^{+}$ T cells in MB49 bearing mice after treated with cisplatin combined with 1-MT (n=6). P-value by two-tailed t-test **(E, F)**. P-value by two-tailed Wilcoxon **(N, P)**. P-value by One-way ANOVA **(M, O)**. All p-value< 0.05 as statistic difference. Error bars represent Mean ± SD, unless otherwise indicated. Three biologically independent experiments were performed **(A, C-L).** Source data are provided as a Source Data file.

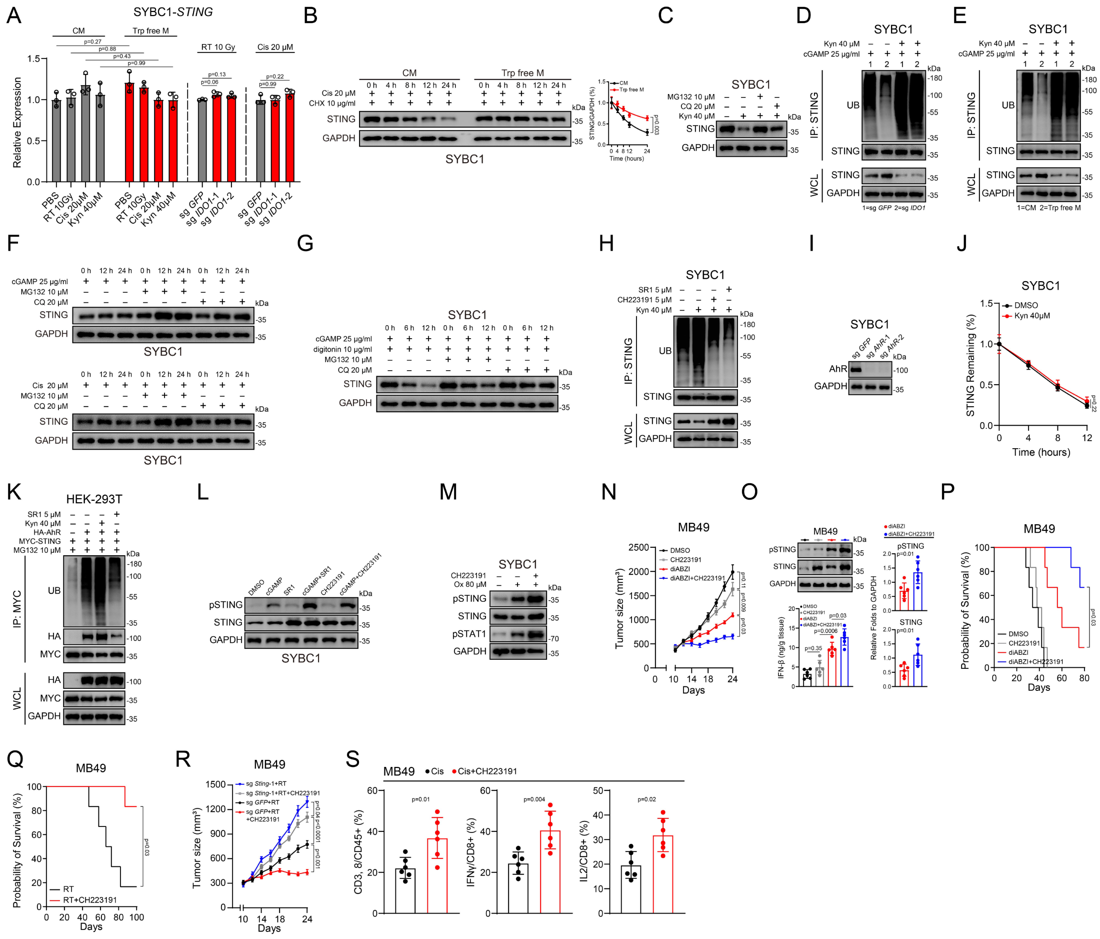

Supplementary Fig.4 Trp-IDO1-Kyn metabolic pathway regulated STING stability through AhR in a ubiquitin-proteasome-dependent manner, related to Fig.4.

A mRNA expression of *STING* after indicated treatment and medium. **B** Immunoassay for STING expression after Trp-free medium cultured and treated with cisplatin in different time points. **C** Immunoassay for STING expression after treated with Kyn combined with CQ or MG132. **D** Coimmunoprecipitation and immunoassay for extracts of cell lysate of *IDO1* knocked-out cells by anti-STING antibodies after treated with Kyn and cGAMP. **E** Coimmunoprecipitation and immunoassay for extracts of cell lysate after cultured with complete medium or Trp-free medium by anti-STING antibodies after treated with Kyn and cGAMP. **F** Immunoassay for STING expression after indicated treatment. **G** Immunoassay for STING expression after indicated treatment combined with digitonin. **H** Coimmunoprecipitation and immunoassay for extracts of cell lysate by anti-STING antibodies after treated with Kyn combined SR1 or CH223191. **I** Immunoassay for *AhR* knocked-out efficiency. **J** mRNA expression of *STING* remaining after treated with actinomycin (10μM). **K** Coimmunoprecipitation and immunoassay for extracts of HEK293T cells under indicated treatment and transfected with HA-AhR, MYC-STING by anti-MYC beads. **L** Immunoassay for pSTING and STING expression after treated with SR1 and CH223191 combined with cGAMP. **M** Immunoassay for representative cGAS-STING related genes expression after treated with oxaliplatin combined with CH223191. **N-O** Effect of CH223191 combined with diABZI on MB49 growth (n=6) (Mean ± SEM) (N), pSTING and STING expression and IFN-β production (O) in tumor. **P** Kaplan-Meier survival curves of MB49 bearing mice after treated with CH223191 combined with diABZI (n=6). **Q** Kaplan-Meier survival curves of MB49 bearing mice after treated with CH223191 combined with RT (n=6). **R** Effect of RT combined with CH223191 on MB49 growth

(n=6) (Mean ± SEM). **S** Percentage of $CD8^+$ T cells, $IFN\gamma^+CD8^+$ T cells, and $IL2^+CD8^+$ T cells in MB49 bearing mice after treated with cisplatin combined with CH223191 (n=6). P-value by One-way ANOVA **(A, N, O, R)**. P-value by two-tailed t-test **(B, J)**. P-value by two-tailed Wilcoxon **(O, S)**. P-value by Kaplan-Meier survival analysis **(P, Q)**. All p-value< 0.05 as statistic difference. Error bars represent Mean ± SD, unless otherwise indicated. Three biologically independent experiments were performed **(A-M)**. Source data are provided as a Source Data file.

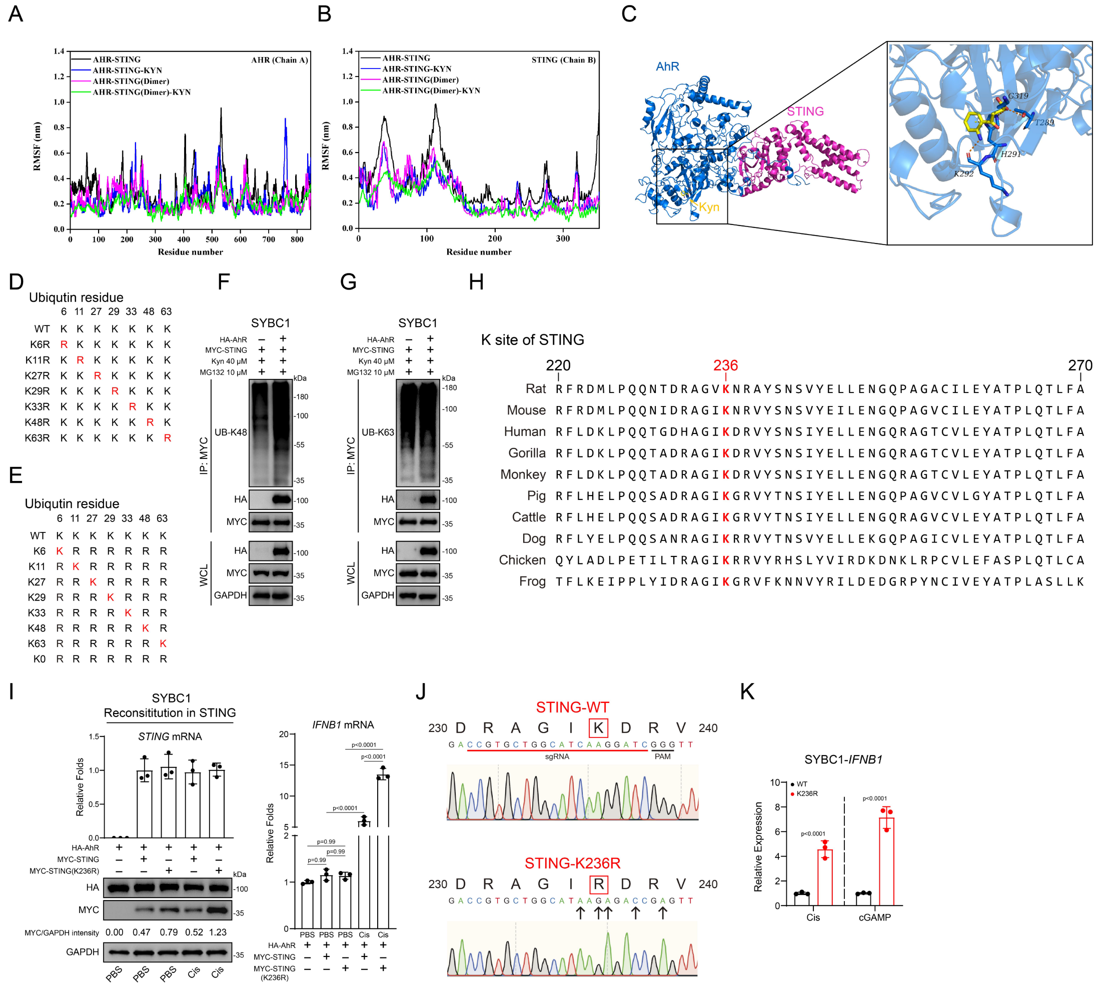

Supplementary Fig.5 STING was ubiquitinated on lysine 236 with K48 linkage by AhR, related to Fig.5.

A, B RMSF plot for AhR-STING, AhR-STING-KYN, AhR-STING (dimer), and AhR-STING-KYN (dimer) of the simulation. **C** The interaction between KYN and AhR for AhR-STING-KYN in the simulation. AhR is colored with marine, STING with magenta, KYN with yellow. The key residues in AhR are shown as marine stick. The orange dashes represent hydrogen bond interaction. The yellow dashes represent π-π conjugation interaction. **D, E** Schematic presentation of UB and its mutants. K0 denotes UB with all lysine residues mutated to arginine. **F, G** Coimmunoprecipitation and immunoassay for extracts of cells lysate after treated with Kyn and transfected with HA-AhR (WT) and MYC-STING (WT) by anti-MYC beads. **H** Alignment of STING amino acid sequences. Highlighted amino acids indicate conserved lysine (K) of STING. **I** Immunoassay and mRNA expression for *STING* and *IFNB1* after transfected with HA-AhR (WT), and MYC-STING (WT), STING (K236R) and treated with cisplatin. P-value< 0.05 as statistic difference by One-way ANOVA (Mean ± SD). **J** Sequencing of parental and individual clones of parental SYBC1 cells with knock-in expression of STING (K236R) mutants. Black arrows indicate mutated nucleotides. A mutated amino acid and its wild-type counterpart are indicated by the solid red box. **K** mRNA expression of *IFNB1* in K236R cells after treated with cisplatin or cGAMP (Mean ± SD). P-value< 0.05 as statistic difference by two-tailed t-test. Three biologically independent experiments were performed **(F, G, I-K)**. Source data are provided as a Source Data file.

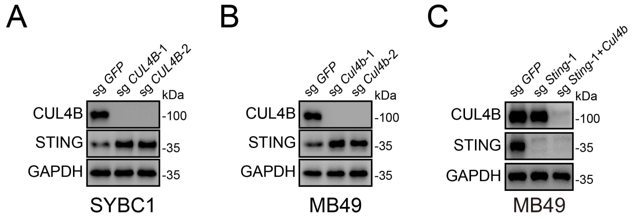

Supplementary Fig.6 AhR worked as an adaptor to facilitate STING ubiquitination through CUL4B-RBX1 E3 complex, related to Fig.6.

A-C Immunoassay for *CUL4B* (A), *Cul4b* (B) and *Sting*/*Cul4b* (C) knocked-out efficiency. Three biologically independent experiments were performed **(A-C).** Source data are provided as a Source Data file.

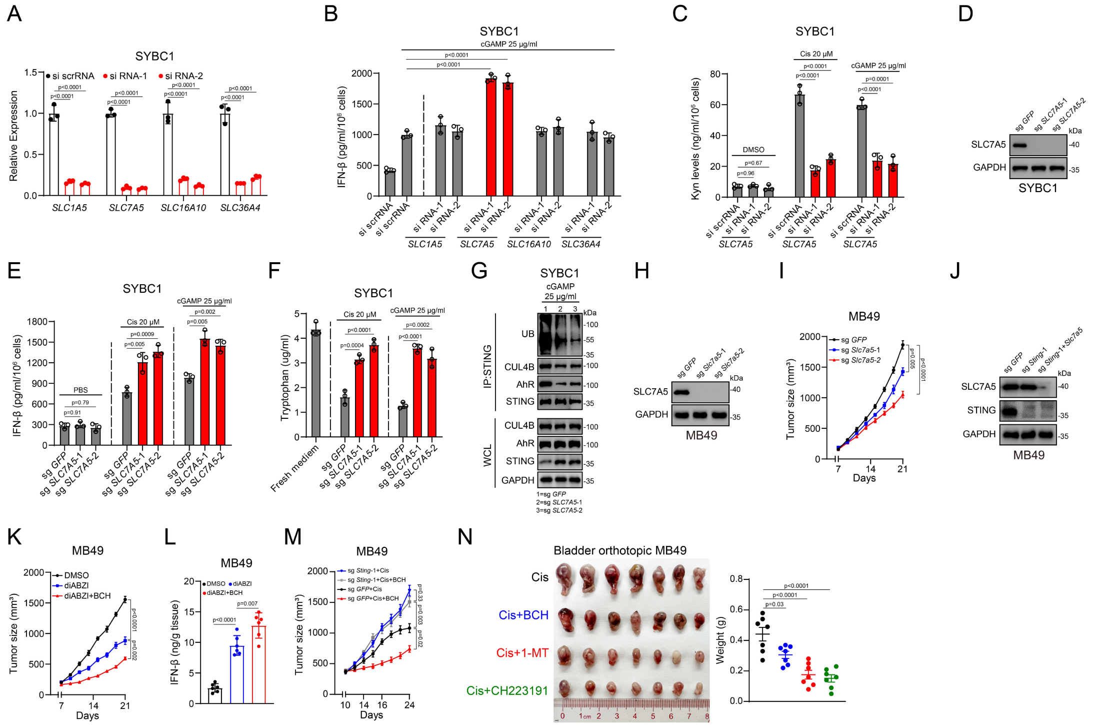

Supplementary Fig.7 SLC7A5 acted as critical Trp transporter to regulate STING stability, related to Fig.7.

A mRNA expression for indicated genes knocked-down efficiency. **B** ELISA for IFN-β content in the supernatant after knocked-down indicated transporters and treated with cGAMP. **C** HPLC-MS detection of Kyn after knocked-down indicated genes and treated with cGAMP or cisplatin. **D** Immunoassay for *SLC7A5* knocked-out efficiency. **E** ELISA for IFN-β content in the supernatant after knocked-out *SLC7A5* and treated with cGAMP or cisplatin. **F** HPLC-MS detection of Trp remaining in the supernatant after knocked-out *SLC7A5* and treated with cGAMP or cisplatin. **G** Coimmunoprecipitation and immunoassay for extracts of cell lysate in *SLC7A5* knocked-out cells and treated with cGAMP by anti-STING antibodies. **H** Immunoassay for *Slc7a5* knocked-out efficiency. **I** Effect of *Slc7a5* knocked-out on MB49 growth (n=6) (Mean ± SEM). **J** Immunoassay for *Sting*/*Slc7a5* knocked-out efficiency. **K, L** Effect of diABZI and BCH on MB49 growth (n=6) (Mean ± SEM) (K) and IFN-β production (L) in tumor. **M** Effect of cisplatin combined with BCH on MB49 growth (n=6) (Mean ± SEM). **N** Effect of BCH, 1-MT, and CH223191 combined with cisplatin on orthotopic MB49 growth (n=7) (Mean ± SEM). P-value by One-way ANOVA **(A-C, E, F, I, K-N)**. All p-value< 0.05 as statistic difference. Error bars represent Mean ± SD, unless otherwise indicated. Three biologically independent experiments were performed **(A-H, J)**. Source data are provided as a Source Data file.

Supplementary Table 1. The binding free energy (in kcal/mol) and its components obtained from the MM/PBSA calculation for AhR-STING, AhR-STING-KYN, AhR-STING (Dimer), and AhR-STING-KYN (Dimer).

Contribution	AhR-STING	AhR-STING-KYN	AhR-STING (Dimer)	AhR-STING-KYN (Dimer)
ΔE_{vdw}	-228.84	-290.83	-245.07	-337.58
ΔE_{ele}	-521.89	-469.01	-504.57	-446.10
ΔG_{polar}	653.51	626.61	641.32	609.93
$\Delta G_{nonpolar}$	-30.15	-38.19	-33.80	-40.92
ΔG_{total}	-127.36	-171.43	-142.13	-214.66

Supplementary Table 2. The contact list between AhR with STING for AhR-STING.

Chain A	Residue	Chain B	Residue	Interaction type
AhR	Tyr371.O	STING	Gln227.NE2	Hydrogen bond
AhR	Asn373.ND2	STING	Thr229.OG1	Hydrogen bond
AhR	Asn373.ND2	STING	Asp237.O	Hydrogen bond
AhR	Gly374.N	STING	Gln227.OE1	Hydrogen bond
AhR	His625.ND1	STING	Asn188.ND2	Hydrogen bond
AhR	His626.O	STING	Arg191.NH1	Hydrogen bond
AhR	Lys628.NZ	STING	Glu249.OE1	Hydrogen bond, Salt bridge
AhR	Lys628.N	STING	Arg253.O	Hydrogen
AhR	Glu633.OE1/OE2	STING	Arg191.NH1/NH2	Hydrogen bond, Salt bridge
AhR	Glu633.OE1	STING	Arg253.NH1	Salt bridge
AhR	Gln640.OE1	STING	Asn187.N	Hydrogen bond
AhR	Gly649.O	STING	Gln227.NE2	Hydrogen bond
AhR	Met650.SD	STING	Gln227.NE2	Hydrogen bond
AhR	Gln658.OE1	STING	Lys224.NZ	Hydrogen bond
AhR	Leu815.N	STING	Asp210.O	Hydrogen bond
AhR	Asn816.O	STING	Gln266.NE2	Hydrogen bond
AhR	Asn817.ND2	STING	Thr263.OG1	Hydrogen bond
AhR	Asn817.ND2	STING	Gln266.OE1	Hydrogen bond
AhR	Asn820.O	STING	His232.NE2	Hydrogen bond
AhR	Leu827.O	STING	Arg94.NH1	Hydrogen bond
AhR	Pro829.CA	STING	Cys91.SG	Hydrogen bond
AhR	His831.N	STING	Arg86.O	Hydrogen bond
AhR	Glu835.OE1	STING	Arg76.NH1/NH2	Hydrogen bond, Salt bridge
AhR	Glu835.OE1	STING	Lys150.NZ	Hydrogen bond, Salt bridge

Supplementary Table 3. The contact list between AhR with STING for AhR-STING-KYN.

Chain A	Residue	Chain B	Residue	Interaction type
AhR	Gln627.OE1	STING	Arg253. NH1	Hydrogen bond
AhR	Gln629.OE1	STING	Arg253.N	Hydrogen bond
AhR	Gln637.NE2	STING	Asp223.OD2	Hydrogen bond
AhR	Lys643.NZ	STING	Asp223.OD1	Hydrogen bond, Salt bridge
AhR	Asn648.OD1	STING	Tyr186.OH	Hydrogen bond
AhR	Asn653.ND2	STING	Gln227.OE1	Hydrogen bond
AhR	Asn804.ND2	STING	Met214.SD	Hydrogen bond
AhR	Asn808.ND2	STING	Asp210.OD1	Hydrogen bond
AhR	Asn808.OD1	STING	Asn211.ND2	Hydrogen bond
AhR	Tyr811.O	STING	Asn211.ND2	Hydrogen bond
AhR	Glu814.N	STING	Asp210.O	Hydrogen bond
AhR	Glu814.OE1/OE2	STING	Lys224.NZ	Hydrogen bond, Salt bridge
AhR	Leu815.N	STING	Asp210.O	Hydrogen bond
AhR	Asn816.ND2	STING	Pro209.O	Hydrogen bond
AhR	Asn816.O	STING	Thr263.OG1	Hydrogen bond
AhR	Leu827.O	STING	Ser162.OG	Hydrogen bond
AhR	Arg837.NH2	STING	Asp301.OD1	Hydrogen bond, Salt bridge
AhR	Pro838.O	STING	Arg14.NH1	Hydrogen bond
AhR	Ser845.OG	STING	Arg14.NE	Hydrogen bond

Supplementary Table 4. The contact list between AhR with STING for AhR-STING (Dimer).

Chain A	Residue	Chain B	Residue	Interaction type
AhR	Tyr371.O	STING	Gln227.NE2	Hydrogen bond
AhR	Asn373.ND2	STING	Thr229.OG1	Hydrogen bond
AhR	Asn373.ND2	STING	Asp237.O	Hydrogen bond
AhR	Asn373.N	STING	Gln227.OE1	Hydrogen bond
AhR	Arg375.NH1/NH2	STING	Asn237.OD1/OD2	Hydrogen bond, Salt bridge
AhR	Ala606.O	STING	Arg191.NE	Hydrogen bond
AhR	His626.O	STING	Arg191.NH1	Hydrogen bond
AhR	Lys628.NZ	STING	Glu249.OE1/OE2	Hydrogen bond, Salt bridge
AhR	Gln629.OE1	STING	Arg191.NH1	Hydrogen bond
AhR	Glu633.OE1/OE2	STING	Arg191.N	Hydrogen bond
AhR	Glu633.OE1	STING	Arg253.NH1	Salt bridge
AhR	Glu633.O	STING	Arg253.NH1	Hydrogen bond
AhR	Gln640.NE2	STING	Leu189.O	Hydrogen bond
AhR	Gly649.O	STING	Gln227.NE2	Hydrogen bond
AhR	Asn655.OD1	STING	Lys224.NZ	Hydrogen bond
AhR	Gln658.OE1	STING	Lys224.NZ	Hydrogen bond
AhR	Pro812.O	STING	Asn211.ND2	Hydrogen bond
AhR	Asn816.ND2	STING	Asp210.O	Hydrogen bond
AhR	Asn816.O	STING	Gln266.NE2	Hydrogen bond
AhR	Asn817.OD1	STING	Thr263.OG1	Hydrogen bond
AhR	Asn817.ND2	STING	Thr263.OG1	Hydrogen bond
AhR	Asn817.ND2	STING	Gln266.OE1	Hydrogen bond
AhR	Thr821.OG1	STING	Gln266.OE1	Hydrogen bond
AhR	Pro833.O	STING	Gln276.NE2	Hydrogen bond
AhR	Ala836.O	STING	Gln276.NE2	Hydrogen bond
AhR	Arg837.NE	STING	Gln273.O	Hydrogen bond

Supplementary Table 5. The contact list between AhR with STING for AhR-STING-KYN (Dimer).

Chain A	Residue	Chain B	Residue	Interaction type
AhR	Gln624.O	STING	Arg191.NH1	Hydrogen bond
AhR	Hie625.ND1	STING	Gly192.N	Hydrogen bond
AhR	Gln627.OE1	STING	Arg253. NH1	Hydrogen bond
AhR	Gln629.OE1	STING	Arg253.N	Hydrogen bond
AhR	Gln629.NE2	STING	Arg253.O	Hydrogen bond
AhR	Gln637.NE2	STING	Asp223.OD2	Hydrogen bond
AhR	Lys643.NZ	STING	Asp223.OD1	Hydrogen bond, Salt bridge
AhR	Asn648.O	STING	Gln227.NE2	Hydrogen bond
AhR	Asn653.ND2	STING	Gln227.OE1	Hydrogen bond
AhR	Lys801.NZ	STING	Gly207.O	Hydrogen bond
AhR	Asn804.ND2	STING	Met214.O	Hydrogen bond
AhR	Asn808.ND2	STING	Asp210.OD2	Hydrogen bond
AhR	Asn808.OD1	STING	Asn211.ND2	Hydrogen bond
AhR	Tyr811.O	STING	Asn211.ND2	Hydrogen bond
AhR	Glu814.N	STING	Asp210.O	Hydrogen bond
AhR	Glu814.OE1/OE2	STING	Lys224.NZ	Hydrogen bond, Salt bridge
AhR	Asn816.ND2	STING	Pro209.O	Hydrogen bond
AhR	Asn816.O	STING	Thr263.OG1	Hydrogen bond
AhR	Arg837.NH2	STING	Asp301.OD1	Hydrogen bond, Salt bridge
AhR	Pro838.O	STING	Arg14.NH1	Hydrogen bond

Supplementary Table 6. Used signature and pathways in this paper.

Teff signature
CD8A,CXCL10,CXCL9,GZMA,GZMB,IFNG,PRF1,TBX21

IFN-Is signaling signature
GOBP_POSITIVE_REGULATION_OF_TYPE_I_INTERFERON_MEDIATED_SIGNALING_PATHWAY

STING signature
WP_CYTOSOLIC_DNASENSING_PATHWAY

Trp metabolism signature
GOBP_TRYPTOPHAN_CATABOLIC_PROCESS_TO_KYNURENINE

Trp transporters signature
GOBP_TRYPTOPHAN_TRANSPORT

AhR activation signature
CRH,ABCG2,SCIN,VAV3,CYP1B1,SORL1,PLA2G4A,ID2,FOXA1,CARD11,SOCS2,GATA3,OVOL1,NQO1,PAX5,KIAA1549,GSTM1,FOS,AQP3,TFF1,HSD17B4,TIPARP,EBF1,AhR,ESR1,FOXQ1,IKZF3,PDS5B,ACTA2,FGFR2,NFE2L2,NCOA2,BLNK,INSIG1,LTBP1,CCL5

Supplementary Table 7. Simulation Model Set.

Systems	Box Dimensions (nm*nm*nm)	Number of Atoms	Number of Water	NaCl Conc. (mol/L)	Time (ns)
AhR-STING	14.9918*15.9165*9.5233	227419	69504	0.15	500
AhR-STING-KYN	14.9793*15.9033*9.5153	227563	69543	0.15	500
AhR-STING (Dimer)	17.2544*18.1980*12.0454	325645	95949	0.15	500
AhR-STING-KYN (Dimer)	17.2319*17.7383*12.0077	319762	93970	0.15	500

Supplementary Table 8. Primer sequences for qRT-PCR, siRNA, and sgRNA assays.

Name	Forward	Reverse
Primer sequences for qRT-PCR		
HLA-A	5’-ACCCTCGTCCTGCTACTCTC-3’	5’-CTGTCTCCTCGTCCCAATACT-3’
CXCL10	5’-GTGGCATTCAAGGAGTACCTC-3’	5’-TGATGGCCTTCGATTCTGGATT-3’
IFNB1	5’-GCTTGGATTCCTACAAAGAAGCA-3’	5’-ATAGATGGTCAATGCGGCGTC-3’
IFNA2	5’-GCTTGGGATGAGACCCTCCTA-3’	5’-CCCACCCCCTGTATCACAC-3’
IFNE	5’-TCCTCAGAAGTCTTTGAGTCCTC-3’	5’-AGGAATTTCTCCGTGTGGTTTTC-3’
IFNK	5’-GTGGCTTGAGATCCTTATGGGT-3’	5’-CAGATTTTGCCAGGTGACTCTT-3’
IFNW1	5'-GAAGGCCCATGTCATGTCTGT-3'	5'-GAGTTGGTCTAGGAGGGTCAT-3'
IFNA1	5'-TCAAAGACTCTCACCCCTGC-3'	5'-CAGTGTAAAGGTGCACATGACG-3'
IFNA4	5'-AGAGGCCGAAGTTCAAGGTTA-3'	5'-ACTGGTGGCCATCAAACTCC-3'
IFNA5	5'-CAAGGTTCAGGGTCACTCAAT-3'	5'-CACCAGGGCCATCAGTAAAAC-3'
IFNA6	5'-ATCTGTTGCTTGGGATGAGAGG-3'	5'-AGGCACAAGGGCTGTACTTTT-3'
IFNA7	5'-CCCACCTCAGGTAGCCTAGTGAT-3'	5'-TCACAGCCCAGAGAGCAGAT-3'
IFNA8	5'-CTGTTCAGCTGTATGGGCAC-3'	5'-GCACAATCAGGGTTGGAGTTC-3'
IFNA10	5'-CACGACGCGTTGAATCAAAAT-3'	5'-ACATTAACCACAATGTAAAGCGAC-3'
IFNA13	5'-TGGTTGAGAACACGGCTCT-3'	5'-CATGTTGGACCAGGTGTTA-3'
IFNA14	5'-CATCTTCGGGATTCCCAATGGC-3'	5'-TTACAGCCCAGAGAGCAGCTT-3'
IFNA16	5'-GGATTCATCTGCTGCTTGGGATG-3'	5'-GAGTCCTCATTCATCAGGGCAA-3'
IFNA17	5'-TGCTGGTGCTCAGCTACAAA-3'	5'-TCCTCCTGGGGAAGTCCAAA-3'
IFNA21	5'-TCCACACTTCTATGACTTCTGCC-3'	5'-TGCCTGCACAGGTAAACATGA-3'
IFNB1 (mouse)	5’-CAGCTCCAAGAAAGGACGAAC-3’	5’-GGCAGTGTAACTCTTCTGCAT-3’

IFNA4 (mouse)	5'-TGATGAGCTACTACTGGTCAGC-3'	5'-GATCTCTTAGCACAAGGATGGC-3'
IFNA2 (mouse)	5'-TACTCAGCAGACCTTGAACCT-3'	5'-CAGTCTTGGCAGCAAGTTGAC-3'
H2-Kb (mouse)	5'-CGTTCCAGGGGATGTACGG-3'	5'-GCTCCCACTTGTGTTTGGTGA-3'
CXCL10 (mouse)	5'-CCAAGTGCTGCCGTCATTTTC-3'	5'-GGCTCGCAGGGATGATTTCAA-3'
TRIF	5'-CCTGGAATCATCATCGGAACAG-3'	5'-TGAGTGGTCTATGGCGTCCT-3'
MAVS	5'-CAGGCCGAGCCTATCATCTG-3'	5'-GGGCTTTGAGCTAGTTGGCA-3'
STING	5'-CACTTGGATGCTTGCCCTC-3'	5'-GCCACGTTGAAATTCCCTTTTT-3'
IL4I1	5'-ACTCGCCCGAAGACATCTAC-3'	5'-CATCCTCGGACATCACGTCTC-3'
IDO1	5'-TCTCATTTCGTGATGGAGACTGC-3'	5'-GTGTCCCGTTCTTGCATTTGC-3'
IDO2	5'-TTCAAGCTCATGTGGACAAGATG-3'	5'-GAGACTTCGACAAATGGAAGGG-3'
TDO2	5'-TCCTCAGGCTATCACTACCTGC-3'	5'-ATCTTCGGTATCCAGTGTCGG-3'
DDC	5'-TGGGGACCACAACATGCTG-3'	5'-TCAGGGCAGATGAATGCACTG-3'
TPH1	5'-TAAGACCTGGGGAACCGTATT-3'	5'-TGGAAAAACCTGTACGCTCTTT-3'
TPH2	5'-CTGCCTCCGAGAAGCAAGAAG-3'	5'-GCATGGAATGGTGGCAAGAG-3'
SLC1A5	5'-CCGCCTTGGCAAGTACATTCT-3'	5'-GGCAGGATGAAACGGCTGA-3'
SLC7A5	5'-CCGTGAACTGCTACAGCGT-3'	5'-CTTCCCGATCTGGACGAAGC-3'
SLC16A10	5'-ATGCTGGAAACCTTCGGCTC-3'	5'-TGAAGACGCTGACTATTGGGC-3'
SLC36A4	5'-CGCGAGGAGCTAGATATGGAT-3'	5'-TGGAAGTCCTAAAAGGCCAGT-3'
Primer sequences for siRNA		
TRIF-1	5'-GCCAGGACAAGCUCUUGUATT-3'	5'-UACAAGAGCUUGUCCUGGCTT-3'
TRIF-2	5'-GGAUCUCUCUAGAGGCAUUTT-3'	5'-AAUGCCUCUAGAGAGAUCCTT-3'
MAVS-1	5'- CUGCCGCAAUUUCAGCAAUTT-3'	5'- AUUGCUGAAAUUGCGGCAGTT-3'
MAVS-2	5'- GCUGUGAGCUAGUUGAUCUTT-3'	5'- AGAUCAACUAGCUCACAGCTT-3'
STING-1	5'- GCCCUUCACUUGGAUGCUUTT-3'	5'- AAGCAUCCAAGUGAAGGGCTT-3'
STING-2	5'- CCGGAUUCGAACUUACAAUTT-3'	5'- AUUGUAAGUUCGAAUCCGGTT-3'

Cullin 4B-1	5’-GCCACGUACCGAUACAGAATT-3’	5’-UUCUGUAUCGGUACGUGGCTT-3’
Cullin 4B-2	5’-GGAGUUAUUUAGGGCUCAUTT-3’	5’-AUGAGCCCUAAAUAACUCCTT-3’
RBX1-1	5’-CUGGGAUAUUGUGGUUGAUTT-3’	5’-AUCAACCACAAUAUCCCAGTT-3’
RBX1-2	5’-GCAGGAACCACAUUAUGGATT-3’	5’-UCCAUAAUGUGGUUCCUGCTT-3’
SLC1A5-1	5’-GCCUUGGCAAGUACAUUCUTT-3’	5’-AGAAUGUACUUGCCAAGGCT-3’
SLC1A5-2	5’-GUCGACCAUAUCUCCUUGATT-3’	5’-UCAAGGAGAUAUGGUCGACTT-3’
SLC7A5-1	5’-GGAAGGGUGAUGUGUCCAATT-3’	5’-UUGGACACAUCACCCUUCCTT-3’
SLC7A5-2	5’-GCAUUAUACAGCGGCCUCUTT-3’	5’-AGAGGCCGCUGUAUAAUGCTT-3’
SLC16A10-1	5’-GCGUCUUCACAGACCUAUUTT-3’	5’-AAUAGGUCUGUGAAGACGCTT-3’
SLC16A10-2	5’-GCAGCAGUGUCUUCACAAUTT-3’	5’-AUUGUGAAGACACUGCUGCTT-3’
SLC36A4-1	5’-CCUGGGAUCACAUCCAAAUTT-3’	5’-AUUUGGAUGUGAUCCCAGGTT-3’
SLC36A4-2	5’-GGGAUUGUUACAACUUUGUTT-3’	5’ACAAAGUUGUAACAAUCCCTT--3’
Primer sequences for sgRNA		
IRF3 (mouse)-1	5’-CACCGTCCAGCTGTGACACCAGCCA-3’	5’-AAACTGGCTGGTGTCACAGCTGGAC-3’
IRF3 (mouse)-2	5’-CACCGGCTGGAAGGCGTGGCCTGGC-3’	5’-AAACGCCAGGCCACGCCTTCCAGCC-3’
STING (mouse)-1	5’-CACCGCCAGCCATCCCACGGCCCAG-3’	5’-AAACCTGGGCCGTGGGATGGCTGGC-3’
STING (mouse)-2	5’-CACCGTGTAGCCCTCATCTTTCTGG-3’	5’-AAACCCAGAAAGATGAGGGCTACAC-3’
IFNAR1 (mouse)-1	5'-CACCGACCCTAAAGTGGAGCAGCCA-3'	5'-AAACTGGCTGCTCCACTTTAGGGT-3'
IFNAR1 (mouse)-2	5'-CACCGCGCCCCGGCCACCAGCACCA-3'	5'-AAACTGGTGCTGGTGGCCGGGGCG-3'
STING-1	5’-CACCGCCATCCATCCCGTGTCCCAG-3’	5’-AAACCTGGGACACGGGATGGATGGC-3’
STING-2	5’-CACCGCCTGCCTGGTGACCCTTTGG-3’	5’-AAACCCAAAGGGTCACCAGGCAGGC-3’
IDO1-1	5’-CACCGGATTTTTATAATGACTGGAT-3’	5’-AAACATCCAGTCATTATAAAAATCC-3’
IDO1-2	5’-CACCGCTGCCTGATCTCATAGAGTC-3’	5’-AAACGACTCTATGAGATCAGGCAGC-3’
STAT1-1	5’-CACCGTCAGACAGTACCTGGCACAG-3’	5’-AAACCTGTGCCAGGTACTGTCTGAC-3’
STAT1-2	5’-CACCGTTATGATGACAGTTTTCCCA-3’	5’-AAACTGGGAAAACTGTCATCATAAC-3’

AhR-1	5’-CACCGTCAAGTCAAATCCTTCCAAG-3’	5’-AAACCTTGGAAGGATTTGACTTGAC-3’
AhR-2	5’-CACCGGGTCCAACTCTGTATTAAGT-3’	5’-AAACACTTAATACAGAGTTGGACCC-3’
Cullin 4B (mouse)-1	5’-CACCGCCTTCGGCGCTCTTGATTGG-3’	5’-AAACCCAATCAAGAGCGCCGAAGGC-3’
Cullin 4B (mouse)-2	5’-CACCGAAAGAGGTGGATAGGGAGGG-3’	5’-AAACCCCTCCCTATCCACCTCTTTC-3’
Cullin 4B-1	5’-CACCGAGCAGCAGCTGAGGGACTGG-3’	5’-AAACCCAGTCCCTCAGCTGCTGCTC-3’
Cullin 4B-2	5’-CACCGGAGGTCAGATCTGCCACTGA-3’	5’-AAACTCAGTGGCAGATCTGACCTCC-3’
SLC7A5 (mouse)-1	5’-CACCGGGGTCACGCCTTCGCCCTCG-3’	5’-AAACCGAGGGCGAAGGCGTGACCCC-3’
SLC7A5 (mouse)-2	5’-CACCGTATCACGCTGCTCAACGGTG-3’	5’-AAACCACCGTTGAGCAGCGTGATAC-3’
SLC7A5-1	5’-CACCGCCCGAAGCGGCGCGCTAG-3’	5’-AAACCTAGCGCGCCGCTTCGGGC-3’
SLC7A5-2	5’-CACCGTCTTCCTTCTCCTCGGCCGC-3’	5’-AAACGCGGCCGAGGAGAAGGAAGAC-3’
Primer sequences for STING1 K236R knock in		
sgRNA for STING1 (K236R)	5′-CCGTGCTGGCATCAAGGATC-3′	
ssODN for STING1 (K236R)	5-’TGCCTGATAACCTGAGTATGGCTGACCCCAACATTCGCTTCCTGGATAAACTGCCCCAGCAGACCGGTGACCGTGC TGGCATaAgaGAcCGaGTTTACAGCAACAGCATCTATGAGCTTCTGGAGAACGGGCAGCGGGTAAGTGTGCAGGGGAG TGGGGGTCTCTGAGGAGGGGTCA-3’	
PCR products amplified	5′-TATCTCCTAGGGCTTCCGCTAGG-3′	5′-TGATCAGATGACACACCCAGAA-3′

Supplementary Table 9. List of antibodies used in this study.

**Antibodies used for Western blotting (WB) and immunoprecipitation (IP).**							
Primary antibodies	Supplier	Catalogue	Application	Host species	Species activity	clone	Dilution
anti-cGAS	Cell Signaling Technology	79978	WB	Rabbit	Hu	N. A	1:2000
anti-STING	Cell Signaling Technology	13647	WB, IP	Rabbit	Hu, Mo	N. A	1:2000 for WB, 5μg for IP
anti-pSTING	Cell Signaling Technology	50907	WB	Rabbit	Hu	N. A	1:2000
anti-pSTING	Cell Signaling Technology	72971	WB	Rabbit	Mo	N. A	1:2000
anti-TBK1	Cell Signaling Technology	38066	WB	Rabbit	Hu	N. A	1:2000
anti-pTBK1	Cell Signaling Technology	5483	WB	Rabbit	Hu	N. A	1:2000
anti-IRF3	Cell Signaling Technology	4302	WB	Rabbit	Hu	N. A	1:2000
anti-pIRF3	Cell Signaling Technology	29047	WB	Rabbit	Hu	N. A	1:2000
anti-STAT1	Cell Signaling Technology	14994	WB	Rabbit	Hu	N. A	1:2000
anti-pSTAT1	Cell Signaling Technology	9167	WB	Rabbit	Hu	N. A	1:2000
anti-γH2AX	Cell Signaling Technology	9718	WB	Rabbit	Hu	N. A	1:2000
anti-IDO1	Cell Signaling Technology	68572	WB	Rabbit	Mo	N. A	1:2000
anti-IDO1	Cell Signaling Technology	86630	WB	Rabbit	Hu	N. A	1:2000
anti-AhR	Cell Signaling Technology	83200	WB	Rabbit	Hu	N. A	1:2000
anti-AhR	R&D systems	AF6697	WB	Sheep	Mo	N. A	1:2000
anti-FLAG	Cell Signaling Technology	14793	WB	Rabbit	Hu	N. A	1:2000
anti-FLAG	Cell Signaling Technology	8146	WB	Mouse	Hu	N. A	1:2000
anti-CUL4B	Proteintech	12916-1-AP	WB	Rabbit	Hu, Mo	N. A	1:2000
anti-RBX1	Cell Signaling Technology	11922	WB	Rabbit	Hu	N. A	1:2000
anti-SLC7A5	Proteintech	28670-1-AP	WB	Rabbit	Hu	N. A	1:2000
anti-SCL7A5	ThermoFisher	PA5-115916	WB	Rabbit	Hu, Mo	N. A	1:2000

anti-GAPDH	Cell Signaling Technology	5174	WB	Rabbit	Hu, Mo	N. A	1:5000
anti-IFNAR1	ThermoFisher	MA5-32006	WB	Rabbit	Hu, Mo	N. A	1:2000
anti-UB	Cell Signaling Technology	43124	WB	Rabbit	Hu	N. A	1:2000
anti-UB	Cell Signaling Technology	3936	WB	Mouse	Hu	N. A	1:2000
anti-HA	Cell Signaling Technology	5017	WB	Rabbit	Hu	N. A	1:2000
anti-HA	Cell Signaling Technology	2367	WB	Mouse	Hu	N. A	1:2000
anti-MYC	Cell Signaling Technology	2272	WB, IP	Rabbit	Hu	N. A	1:2000 for WB, 5µg for IP
anti-MYC	Cell Signaling Technology	2276	WB, IP	Mouse	Hu	N. A	1:2000 for WB, 5µg for IP
anti-UBK48	Cell Signaling Technology	12805	WB	Rabbit	Hu	N. A	1:2000
anti-UBK63	Cell Signaling Technology	12930	WB	Rabbit	Hu	N. A	1:2000
IgG control Polyclonal antibody	Proteintech	30000-0-AP	IP	Rabbit	Hu	N. A	5µg for IP
pierce anti-c-Myc magnetic beads	ThermoFisher	88842	IP	Mouse	Hu	N. A	20ul per test for IP
anti-MYC nanobody magarose beads	AlpalifeBio	KTSM1336	IP	Alpaca	Hu	N. A	25ul per test for IP
anti-mouse IgG, HRP-linked Antibody	Cell Signaling Technology	7076	WB	Horse	Mo	N. A	1:5000
anti-rabbit IgG, HRP-linked Antibody	Cell Signaling Technology	7074	WB	Goat	Rabbit	N. A	1:5000
HRP-conjugated Affinipure Rabbit anti-Sheep IgG(H+L)	Proteintech	SA00001-16	WB	Rabbit	Sheep	N. A	1:5000

Antibodies used for flow cytometric analysis.							
Primary antibodies	Supplier	Catalogue	Application	Host species	Species activity	clone	Dilution
AF700 anti-human CD326	Biolegend	324244	Fc	mouse	Hu	9C4	5ug per test
BV605 anti-human CD45	Biolegend	368524	Fc	mouse	Hu	2D1	5ug per test
BV650 anti-mouse CD45	Biolegend	103151	Fc	Rat	Mo	30-F11	5ug per test
PC7 anti-mouse CD3	Biolegend	100220	Fc	Rat	Mo	17A2	5ug per test
FITC anti-mouse CD8	Biolegend	100705	Fc	Rat	Mo	53-6.7	5ug per test
PE anti-mouse IFNG	Biolegend	505807	Fc	Rat	Mo	XMG1.2	5ug per test
APC anti-mouse IL2	Biolegend	503809	Fc	Rat	Mo	JES6-5H4	5ug per test
**Antibodies used for immunofluorescence (IF).**							
Primary antibodies	Supplier	Catalogue	Application	Host species	Species activity	clone	Dilution
AhR	ThermoFisher	MA1-513	IF	Mouse	Hu	RPT9	1:100
STING	Proteintech	66680-1-Ig	IF	Mouse	Hu	1F1E1	1:100
CUL4B	Proteintech	12916-1-AP	IF	Rabbit	Hu	N. A	1:100
**Antibodies used for Neutralizing antibody.**							
Primary antibodies	Supplier	Catalogue	Application	Host species	Species activity	clone	Dilution
anti-mouse IFNAR1	Selleck	A2121	Neutralizing	N. A	Mo	MAR1-5A3	100μg per test
anti-mouse CD8α	Bioxcell	BE0061	Neutralizing	N. A	Mo	53-6.7	100μg per test

Supplementary References

1. Sjodahl G, *et al.* Different Responses to Neoadjuvant Chemotherapy in Urothelial Carcinoma Molecular Subtypes. *Eur Urol* **81**, 523-532 (2022).

2. Seiler R, *et al.* Impact of Molecular Subtypes in Muscle-invasive Bladder Cancer on Predicting Response and Survival after Neoadjuvant Chemotherapy. *Eur Urol* **72**, 544-554 (2017).
