# Supplementary material for: AhR diminishes the efficacy of chemotherapy via suppressing STING dependent type-I interferon in bladder cancer
Source: Nat Commun. 2023 Sep 5;14:5415. doi: 10.1038/s41467-023-41218-5 (PMC10480448; doi:10.1038/s41467-023-41218-5)
Supplement: Supplementary file 3 — Description of Additional Supplementary Files [file 41467_2023_41218_MOESM3_ESM.pdf]

Description of Additional Supplementary Files

Title: **Supplementary Data 1**

Description: I-TASSER Results for AhR & initial and final configurations for molecular dynamics trajectories

Title: **Supplementary Movie 1**

Description: Interaction of monomer STING with AhR in state without endogenous ligand (Kyn) binding.

Title: **Supplementary Movie 2**

Description: Interaction of monomer STING with AhR in state with endogenous ligand (Kyn) binding.

Title: **Supplementary Movie 3**

Description: Interaction of dimer STING with AhR in state without endogenous ligand (Kyn) binding.

Title: **Supplementary Movie 4**

Description: Interaction of dimer STING with AhR in state with endogenous ligand (Kyn) binding.
