# Supplementary material for: AhR diminishes the efficacy of chemotherapy via suppressing STING dependent type-I interferon in bladder cancer
Source: Nat Commun. 2023 Sep 5;14:5415. doi: 10.1038/s41467-023-41218-5 (PMC10480448; doi:10.1038/s41467-023-41218-5)
Supplement: Supplementary file 9 — Reporting Summary [file 41467_2023_41218_MOESM9_ESM.pdf]

nature portfolio

Corresponding author(s): zhuowei Liu

Last updated by author(s): Aug 25, 2023

Reporting Summary

Nature Portfolio wishes to improve the reproducibility of the work that we publish. This form provides structure for consistency and transparency in reporting. For further information on Nature Portfolio policies, see our Editorial Policies and the Editorial Policy Checklist.

Statistics

For all statistical analyses, confirm that the following items are present in the figure legend, table legend, main text, or Methods section.

n/a	Confirmed	
☐	☒	The exact sample size (*n*) for each experimental group/condition, given as a discrete number and unit of measurement
☐	☒	A statement on whether measurements were taken from distinct samples or whether the same sample was measured repeatedly
☐	☒	The statistical test(s) used AND whether they are one- or two-sided<br>*Only common tests should be described solely by name; describe more complex techniques in the Methods section.*
☐	☒	A description of all covariates tested
☐	☒	A description of any assumptions or corrections, such as tests of normality and adjustment for multiple comparisons
☐	☒	A full description of the statistical parameters including central tendency (e.g. means) or other basic estimates (e.g. regression coefficient) AND variation (e.g. standard deviation) or associated estimates of uncertainty (e.g. confidence intervals)
☐	☒	For null hypothesis testing, the test statistic (e.g. *F*, *t*, *r*) with confidence intervals, effect sizes, degrees of freedom and *P* value noted<br>*Give P values as exact values whenever suitable.*
☒	☐	For Bayesian analysis, information on the choice of priors and Markov chain Monte Carlo settings
☒	☐	For hierarchical and complex designs, identification of the appropriate level for tests and full reporting of outcomes
☐	☒	Estimates of effect sizes (e.g. Cohen's *d*, Pearson's *r*), indicating how they were calculated

Our web collection on statistics for biologists contains articles on many of the points above.

Software and code

Policy information about availability of computer code

Data collection	FASC: cytoFLEX LX; Real-time PCR: Bio-Rad CFX96; LC/MS: Q-Exactive mass spectrometer.
Data analysis	GraphPad Prism version 9 and spss24 were used for data analysis; FACS data were analyzed with FlowJo version 10; Pathways gene enrichment were analyzed by using GSEA v4.0.3; Immunohistochemical Staining and Scoring: AxioVision Rel.4.6 computerized image analysis system and HALO V3; Raw LC-MS data was analyzed with MZmine 2.5.3

For manuscripts utilizing custom algorithms or software that are central to the research but not yet described in published literature, software must be made available to editors and reviewers. We strongly encourage code deposition in a community repository (e.g. GitHub). See the Nature Portfolio guidelines for submitting code & software for further information.

Data

Policy information about availability of data

All manuscripts must include a data availability statement. This statement should provide the following information, where applicable:

- Accession codes, unique identifiers, or web links for publicly available datasets
- A description of any restrictions on data availability
- For clinical datasets or third party data, please ensure that the statement adheres to our policy

All data supporting the findings of this study are available within the Article, Supplementary Information or Source Data file. All original data for this study can be obtained from the corresponding author. TCGA datasets for BLCA, were all obtained from UCSC Xena (https://xenabrowser.net/datapages/). BLCA cohort with neoadjuvant chemotherapy datasets were obtained from Gene Expression Omnibus (https://www.ncbi.nlm.nih.gov/geo/, accession numbers: GSE169455 and

GSE87304) or supplementary material to the article (https://www.nature.com/articles/s41467-020-18640-0). Initial and final configurations for molecular dynamics trajectories refer to Supplementary Data 1. This paper does not report original code. Source data are provided with this paper.

Research involving human participants, their data, or biological material

Policy information about studies with human participants or human data. See also policy information about sex, gender (identity/presentation), and sexual orientation and race, ethnicity and racism.

Reporting on sex and gender	The gender information of patients with bladder cancer were retrospectively collected through the medical record.
Reporting on race, ethnicity, or other socially relevant groupings	The relevent information of patients with bladder cancer were retrospectively collected through the medical record.
Population characteristics	All samples were pathologically confirmed as urothelial carcinoma of the bladder.
Recruitment	All samples were obtained from the Sun Yat-sen University Cancer Center, Sun Yat-sen University, Guangzhou. All patients were pathologically diagnosed with bladder cancer and collected with informed consent.
Ethics oversight	The Institutional Ethical Review Boards of Sun Yat-sen University Cancer Center approved this study.

Note that full information on the approval of the study protocol must also be provided in the manuscript.

Field-specific reporting

Please select the one below that is the best fit for your research. If you are not sure, read the appropriate sections before making your selection.

[x] Life sciences [ ] Behavioural & social sciences [ ] Ecological, evolutionary & environmental sciences

For a reference copy of the document with all sections, see nature.com/documents/nr-reporting-summary-flat.pdf

Life sciences study design

All studies must disclose on these points even when the disclosure is negative.

Sample size	5 BC samples were collected for flow cytometry analysis .
Data exclusions	No data were excluded from the analyses.
Replication	As reported in the figure legends, experiments were performed at least three times with similar results, the findings were reliably reproduced.
Randomization	For all in vivo experiments, animals were randomly assigned into a treatment group after tumor inoculation (n=6). The starting tumor burden in the treatment and control groups was similar before treatment.
Blinding	The investigators were not blinded to sample allocation during experiment and outcome assessment, because results used were obtained using objective quantitative methods.

Reporting for specific materials, systems and methods

We require information from authors about some types of materials, experimental systems and methods used in many studies. Here, indicate whether each material, system or method listed is relevant to your study. If you are not sure if a list item applies to your research, read the appropriate section before selecting a response.

Materials & experimental systems

n/a	Involved in the study	
[ ]	[x]	Antibodies
[ ]	[x]	Eukaryotic cell lines
[x]	[ ]	Palaeontology and archaeology
[ ]	[x]	Animals and other organisms
[x]	[ ]	Clinical data
[x]	[ ]	Dual use research of concern
[x]	[ ]	Plants

Methods

n/a	Involved in the study	
[x]	[ ]	ChIP-seq
[ ]	[x]	Flow cytometry
[x]	[ ]	MRI-based neuroimaging

Antibodies

Antibodies used	The antibodies used are listed in Supplementary Table 9.
Validation	All antibodies were validated by immune blotting and immunofluorescence imaging prior to isotope-polymer conjugation Antibodies were tested for cell type and inter-cell location specificity within positive control tissues

Eukaryotic cell lines

Policy information about cell lines and Sex and Gender in Research

Cell line source(s)	Human cells (including UMUC-3, Hela, MCF-7, HEK293T cells) and mouse tumor cells (including MB49 cells) were obtained from ATCC. SYBC1 generated in-house.
Authentication	STR fingerprint analysis
Mycoplasma contamination	All cell lines in our laboratory are routinely tested for mycoplasma contamination and cells used in this study are negative for mycoplasma.
Commonly misidentified lines (See ICLAC register)	No cell line used in the paper is listed in ICLAC database.

Animals and other research organisms

Policy information about studies involving animals; ARRIVE guidelines recommended for reporting animal research, and Sex and Gender in Research

Laboratory animals	For the in vivo part of the study, 6- to 8-week-old female C57BL/6 mice were purchased from the Guangdong Medical Laboratory Animal Center (Foshan, China). These animals were maintained under defined conditions at the Animal Experiment Center of Sun Yat-Sen University. AhR flox/flox mice were bred to lyze-Cre mice to generate mice with specific AhR deletion in myloid cells. All mice were maintained under specific pathogen free conditions (temperature of 20 ~ 26℃, daily temperature difference ≤3℃, relative humidity of 40% ~ 70%, fresh air exchange 10 times/h, air velocity ≤0.18m/s, pressure difference 25Pa, cleanliness level 10,000, ammonia concentration 15mg/m³, noise ≤60dB, illumination 150-300Lux.).
Wild animals	The study did not involve wild animals.
Reporting on sex	The study did not involve.
Field-collected samples	The study did not involve samples collected from field.
Ethics oversight	All animal experiments were approved by the Animal Care and Use Committee of Sun Yat-Sen University

Note that full information on the approval of the study protocol must also be provided in the manuscript.

Flow Cytometry

Plots

Confirm that:

- [x] The axis labels state the marker and fluorochrome used (e.g. CD4-FITC).
- [x] The axis scales are clearly visible. Include numbers along axes only for bottom left plot of group (a 'group' is an analysis of identical markers).
- [x] All plots are contour plots with outliers or pseudocolor plots.
- [x] A numerical value for number of cells or percentage (with statistics) is provided.

Methodology

Sample preparation	The sample preparation was described in the methods section.
Instrument	cytoFLEX LX
Software	FlowJo version 10.
Cell population abundance	When cells were sorted or enriched, the purity was confirmed by flow cytometry and in each case was above 90% purity.
Gating strategy	The gating strategy were provided in Fig. 1I, Supplementary Figure 1L

- [x] Tick this box to confirm that a figure exemplifying the gating strategy is provided in the Supplementary Information.
